# Design and utilization of the colorectal and pancreatic neoplasm virtual biorepository: An early detection research network initiative

**DOI:** 10.4103/2153-3539.70831

**Published:** 2010-10-01

**Authors:** Waqas Amin, Harpreet Singh, Lynda Ann Dzubinski, Robert E. Schoen, Anil V. Parwani

**Affiliations:** 1 Department of Biomedical Informatics, University of Pittsburgh Medical Center, Pittsburgh, PA, USA; 2 Department of Pathology, University of Pittsburgh Medical Center, Pittsburgh, PA, USA; 3 Department of Gastroenterology, Hepatology and Nutrition, University of Pittsburgh Medical Center, Pittsburgh, PA, USA

**Keywords:** Colorectal and pancreatic neoplasm, tissue banking informatics

## Abstract

**Background:**

The Early Detection Research Network (EDRN) colorectal and pancreatic neoplasm virtual biorepository is a bioinformatics-driven system that provides high-quality clinicopathology-rich information for clinical biospecimens. This NCI-sponsored EDRN resource supports translational cancer research. The information model of this biorepository is based on three components: (a) development of common data elements (CDE), (b) a robust data entry tool and (c) comprehensive data query tools.

**Methods:**

The aim of the EDRN initiative is to develop and sustain a virtual biorepository for support of translational research. High-quality biospecimens were accrued and annotated with pertinent clinical, epidemiologic, molecular and genomic information. A user-friendly annotation tool and query tool was developed for this purpose. The various components of this annotation tool include: CDEs are developed from the College of American Pathologists (CAP) Cancer Checklists and North American Association of Central Cancer Registries (NAACR) standards. The CDEs provides semantic and syntactic interoperability of the data sets by describing them in the form of metadata or data descriptor. The data entry tool is a portable and flexible Oracle-based data entry application, which is an easily mastered, web-based tool. The data query tool facilitates investigators to search deidentified information within the warehouse through a “point and click” interface thus enabling only the selected data elements to be essentially copied into a data mart using a dimensional-modeled structure from the warehouse’s relational structure.

**Results:**

The EDRN Colorectal and Pancreatic Neoplasm Virtual Biorepository database contains multimodal datasets that are available to investigators via a web-based query tool. At present, the database holds 2,405 cases and 2,068 tumor accessions. The data disclosure is strictly regulated by user’s authorization. The high-quality and well-characterized biospecimens have been used in different translational science research projects as well as to further various epidemiologic and genomics studies.

**Conclusions:**

The EDRN Colorectal and Pancreatic Neoplasm Virtual Biorepository with a tangible translational biomedical informatics infrastructure facilitates translational research. The data query tool acts as a central source and provides a mechanism for researchers to efficiently query clinically annotated datasets and biospecimens that are pertinent to their research areas. The tool ensures patient health information protection by disclosing only deidentified data with Institutional Review Board and Health Insurance Portability and Accountability Act protocols.

## INTRODUCTION

Recent developments in translational research have provided increased opportunities for the identification of new cancer biomarkers. This approach has generated a demand for biorepositories that are capable of providing quality-controlled biospecimens with standardized clinical and pathology annotation. The success of biorepository depends on informatics architecture and controlled vocabulary and ontology that provides semantic and syntactic operability for a robust system of clinical annotation that allows samples to be better matched to the research community requirements.[1,2] Over the last decade, the implementation of tissue banking and informatics systems has been recognized as basic entities in the development of a translational research initiative. The general consensus and the recommendation from the Research and Development (RAND) Corporation’s Case Study of Existing Human Tissue repositories, state that “…the collection of consistent and high quality data associated with every biospecimen and employing a standardized set of common data element for annotation…” is now broadly considered best practice, which reflects the need for such standardization.[3–5]

The University of Pittsburgh’s Early Detection Research Network (EDRN) Colorectal and Pancreatic Neoplasm Virtual Biorepository was funded by the National Cancer Institute in 2001. The main objective of this initiative was to gather dispersed biospecimens and data pertaining to patients who have been regularly managed and followed-up from 1990 to 2000 either for regular colorectal cancer screening or gastrointestinal malignancies, premalignant conditions, inflammatory bowel disease, pancreatic diseases and pancreatic and colorectal cancers at different centers in the University of Pittsburgh Medical Center (UPMC) hospital networks. Prospective patients are also enrolled in this project since 2001 and all patients are monitored and followed on a continuous basis. The EDRN Colorectal and Pancreatic Neoplasm database is a bioinformatics system that integrates data from various clinical, pathologic and molecular resources into one design facilitated by a set of common data elements (CDEs) that facilitates multimodal datasets to be better matched, understandable and sharable across various hospital data resources. Development of CDEs was defined by mutual consensus among professionals using the North American Association of Central Cancer Registries (NAACCR) standards, American Joint Committee on Cancer (AJCC) staging manual_and College of American Pathologists (CAP) cancer protocols and checklists.[6–8]

The EDRN Colorectal and Pancreatic Neoplasm Virtual Biorepository is a web-based clinical annotation warehouse system that is supported by Oracle-based three-tiered architecture. The data-warehouse provides data entry, data mining and data analysis tools for clinicopathology datasets. The query tool accesses the database through a highly constrained “click and point” interface and provides data that is collected over a period of time. This enables researchers and clinician to better understand the disease process, progression, recurrence and outcome.

## METHODS

The key objective of this initiative is to design and develop a colorectal and pancreatic neoplasm virtual bioreposity that provides high-quality clinically annotated colorectal and pancreatic neoplasm biospecimens for the support of translational research. There are various issues at every step of the way, which have been dealt with meticulously to ensure quality and maintenance of protocol. These issues include participant inclusion, patient consent, patient clinical follow-up and data collection, biospecimen accession and maintenance of accession standards, specimen handling, storage and inventory, data annotation and entry, data deidentification, quality assurance of database and tissue inventory, tissue distribution and data access for researchers. These issues have been discussed in greater detail in the following sections and in the discussion portion.

### Patient Participation Criteria

All colorectal and pancreatic cancer patients are registered and consented by the research nurse coordinators (RNCs) at a physician office. The criteria to include the patient in the study are the following:

- Age more than or equal to18 years
- The participants will either have or be suspected to have digestive disorder/diseases
- Participants must be able to give informed consent

The patient consent and Institutional Review Board (IRB) document are maintained by the RNCs, and if any patient withdraws from the research, the RNC immediately informs the data administrator who removes the relevant data to the withdrawn case from the database and declares the cases closed in the database. If the relevant data and specimen of the off-study case has already been given to the researcher then no further updated data and specimen are given to researchers.

### Patient Health Information Protection and Deidentification Process

The EDRN Colorectal and Pancreatic Neoplasm Virtual Biorepository uses biospecimens and data collected at the UPMC. All specimens are accrued by standardized IRB-approved protocols. Each consented case is entered into the database by the RNC and relevant clinical and pathological data are stored by data mangers and pathologists. The EDRN database holds patient-identified data but secures patient’s privacy by following the Health Insurance Portability and Accountability Act (HIPAA)-approved protocols. In order to protect patient privacy, each case is assigned with an automated generated 12 digit deidentified case identification number and patient identifiers (18 data elements identified by HIPAA) are secured by the database. Thus, queries of publicly available websites generate deidentified datasets for the research community (the so-called “safe harbor” approach to HIPAA compliance).[9]

The deidentification process is performed by an honest broker (those are designated as RNC), tissue bank technician, data mangers and pathologist for this project. An honest broker acts as a barrier between the clinical environment that contains identified confidential patient information and the research community in which all patient health information must be deidentified. In reality, the honest broker is the only person or organization that provides a linkage between research identifiers and clinical identifiers. The honest broker also performs as an independent third party to take responsibility and control of the deidentification process and to keep patient health information identifiers (patient’s name, date of birth, social security numbers and medical record numbers) in a separate log and avoid linking them to research deidentifiers in the database.

**Figure 1:**
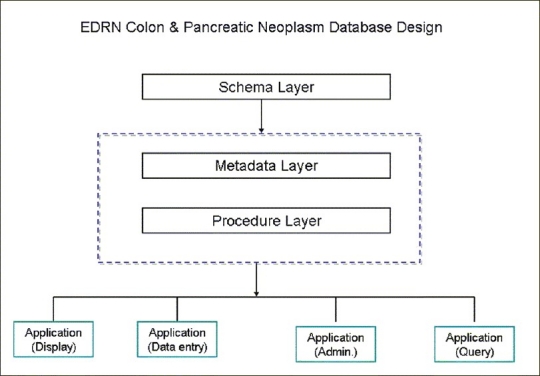
An overview of the early detection research network colon and pancreatic virtual biorepository system designed for central database as a multi-tiered application using Oracle 9i

An honest broker is an individual, organization or system acting on behalf of the covered entity to collect and provide health information to the investigators in such a way that it would not be logically possible for the investigators or others to identify the corresponding patients directly or indirectly. The honest broker cannot be one of the investigators. A researcher may use the services of an honest broker system to obtain the Protected Health Information in a deidentified manner. Deidentification means that the patient-subjects cannot be identified by researchers or others directly or indirectly through identifiers linked to the patient-subject. This honest broker system/service will deidentify medical record information by automated and/or manual methods. All honest broker systems are approved in advance by both the IRB of record and the UPMC. If an honest broker system is not part of the UPMC-covered entity, a valid business associate agreement with UPMC is executed with the UPMC in order to access the UPMC-held Protected Health Information for deidentification. If an honest broker system is to be used to obtain deidentified protected health information, this fact must be identified in the study’s IRB submission. The honest brokers are individuals who have clinical responsibilities as tissue bankers in the Health Sciences Tissue Bank (HSTB), postdoctoral fellows responsible for managing the pathology data or as a cancer registry specialist in the UPMC registry information services. Based on their clinical job duties, educational backgrounds and experiences vary, which depends on the nature of the projects, and these individuals can work autonomously or collaboratively to meet biospecimen and/or data needs [Figure 1].[10]

### Development of Data Collection Standards

The development of CDE for the Colorectal and Pancreatic Neoplasm Virtual Biorepository is performed by mutual consensus of the various domain experts, including gastroenterologists, gastrointestinal oncologists/pathologists, researchers, cancer registrars and incorporating data collection standards from open-source sources, NAACCR, AJCC staging manual and CAP.[6–8] The informatics-supported system allows the CDEs that are used for annotating the tissue samples to be semantic and syntactic and interoperable across different hospital systems by describing them in the form of metadata or data descriptors.

Each common data element is linked to an object or concept, attribute and valid value(s). Specifically for each of the approved CDEs, the data collectors need to know (1) the fundamental definition of the data element (i.e., date of diagnosis), (2) how that data element will be collected, (3) what are the consensus acceptable values or codes for the data element and (4) what is the acceptable data format for inclusion into the central database. Although the concept of formalized metadata is fairly straight forward, it has been rarely incorporated by clinical and research groups building databases.[11]

### Building a Data Collection Application and Common Methods for Data Transmission

After agreement on CDEs by the coordinating committee of Colorectal and Pancreatic Neoplasm Virtual Biorepositoy, the development of a data entry application using Microsoft Access is generated. The resource has also designed a CDE data dictionary to provide guidelines to the data managers about the definition of each CDE, its valid values, variable constraints, validation rules and any requirements and useful comments with regard to each of the CDEs. It also explains the inclusion criteria of the types of biospecimens collected for the resource. This data dictionary is a dynamic document that is freely available and is regularly updated and refined as specific information or issues arise.[12] The resource developed its database using Oracle software, and data are transferred to the database by an inbuilt data entry tool. The data is captured as identified. However, after the deidentification process by the Microsoft Access application, the data is displayed as deidentified on a web-based query tool in accordance with the HIPAA compliance.

### Collection of Data and Management

The RNC is responsible for collecting the demographic and epidemiological data. The surgical pathology report and all histological sections available by the gastrointestinal pathologist are reviewed to accurately categorize each case. The pathologist then selects representative slides and paraffin blocks according to a standardized HSTB protocol. The selected slides show specific features of the case likely to be of interest to scientific investigators. Specific data elements are collected on these slides. After the pathological data is reviewed, the certified tumor registrars review and extract clinical data for cases accrued into the database. Data is collected and annotated using common web-based data entry forms that are correlated with the CDEs developed within the database.[13]

### Data Quality Assurance (QA) of Collected Data

After importing the data into the Colorectal and Pancreatic Neoplasm database, the data is processed using policies, variable constraints and logistical tests established by the resource. The QA checks are carried out to spot any missing essential CDE data or possible data input errors, including field and cross-field checking. The valid field options, defined for each data element in the CDE dictionary, are checked using these automated QA measures. Accepted records are then uploaded into the resource database and records and discrepancies are returned to the data collecting source for review and correction. The data manager documents the reasons for rejection.[14]

The resource assembles a large number of CDEs, a subset of the data that is collected by cancer registrars through review of in- and out-patient clinical records. The resource has established an audit review system that allows verifying the collected data from the primary source clinical record. In this audit review system, the data manger selects 5% of the newly entered cases to be reexamined by honest brokers, cancer registrar and data mangers. On completion of their review, the audit reviewers submit a report of their findings and their recommendations to the project coordinating committee. The QA team discusses their findings in the next general meeting of the project coordinating committee and makes plans to implement the proposed recommendations. To reduce the chance of providing redundant data, a final review of the associated case CDEs for possible errors and an update of the clinical information is performed to guarantee the most accurate and up-to-date information for requesting investigators.[14]

All pathologic CDEs related to the specimen cases are entered after complete specimen review by trained gastroenterology pathologists. In the independent review process, a series of randomly selected cases are rereviewed. The data manager or pathologist randomly selects cases for independent review from those added to the resource within certain cut-off dates. The independent review material consists of two to five matrix slides for each case. Once received, the pathologists review and annotate the pathologic matrix and histology CDE data for the case using their established processes. They analyze for observer variability and diagnostic error rates. This process occurs at regular intervals twice a year and is established to check specimen resource quality. Any discrepancies identified through independent review are communicated to the pathology subcommittee. The pathology subcommittee then discusses their findings in the subsequent general meeting of the Coordinating Committee through a formal report with recommendations for changes in process, as indicated by the independent review findings. Any errors discovered during the independent review process are corrected.[14]

### Development of the EDRN Colon and Pancreatic Cancer Database

#### Informatics architecture and integration

The EDRN colorectal and pancreatic database is designed as a multi-tiered application using the Oracle 10g database server, Oracle 10g application server and mod pl/sql, also known as pl/sql server. This approach was adopted previously in the development of the Pennsylvania Alliance of Bioinformatics Consortium (http://pcabc.upmc.edu/main.cfm), Cooperative Prostate Cancer Tissue Resource (http://www.cpctr.info) and SPOREs Head and Neck Neoplasm Virtual Biorepository Repository.

**Figure 2:**
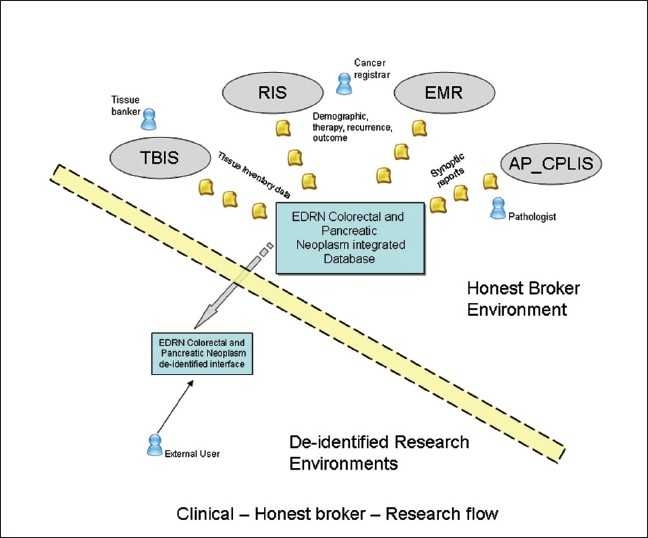
Work flow data collection and transfer at end user interface in the clinical and research environments

The information model of the database consists of *Schema layer* – defines actual data and data relations. All classified data are stored in numbers and keys. *Metadata layer* – in which all data is defined in terms of data elements and “groups of data elements.” Data descriptions such as data attributes, display attributes, valid values, DB Link, validation rules and documentation are supported in Meta data. The Meta data layer defines the application layer. *Procedures/function layer* – is a set of dynamic procedures/functions (in PL/SQL or Java) with control data transformation at the back end. The procedures accommodate changes in the Meta data and immediately reflect the changes in the application layer. *Application layer (Form builder)* – is a set of “applications,” including metadata dictionary builder and manager, user management, data entry/transfer, query, display, etc. Depending on the user privileges, the appearance will be different. These differences are driven by the Meta data and user management [Figure 2].

## RESULTS

**Figure 3:**
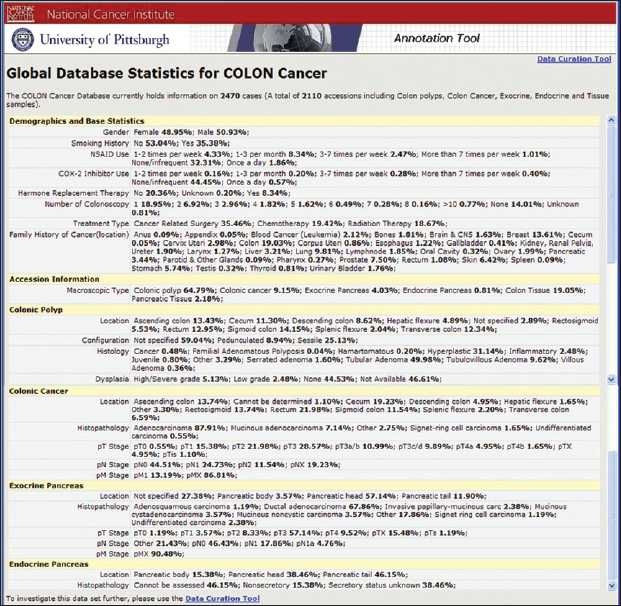
The early detection research network colon and pancreatic virtual biorepository statistics on colon and pancreatic tissue collection

The EDRN Colorectal and Pancreatic Neoplasm Virtual Biorepository has proven itself to be a very valuable and rich resource for researchers. Since October 2001, the EDRN database currently holds information on 2,470 prospective cases. For all these cases, blood samples have also been accrued. The purpose of patient participation is to ensure early detection and screening for colorectal and pancreatic diseases in order to prevent the progression of malignancies as well as the collection of well-annotated biospecimens consisting of benign, premalignant and malignant cases for facilitation of further basic and clinical science research. A total of 2,110 accessions including colon polyps, colon cancer, exocrine, endocrine pancreatic cancers and tissue samples from rare benign to premalignant colorectal and pancreatic pathologies were included [Figure 3]. In addition, the resource contains valuable clinicopathology information on benign colorectal and pancreatic pathologies, such as inflammatory bowel disease with dysplasia, diverticulitis, Whipple’s disease, angiodysplasia and many more.

The overall workflow of the system for entering data into the virtual biorepository will be performed as follows. Currently, this system is supported with the Oracle database Server 10.2.0.2 and Oracle Application Server 10.1.0.2. Both the database server and the application server are configured as virtual hosts on the IBM Power 6 Series 570 hardware. The Oracle database partition is running AIX 5L and is configured with 4 CPUs and 6 GB of RAM. The application server is running Windows 2003 Server SP2 and is configured with 1 CPU and 1.26 GB of RAM. All these hardware and system software are centrally supported for backup and routine maintenance by the Informatics Services Division. The Oracle database administrator (DBA) maintains the database-related services. Web-based application database repository query tools, data entry annotations and user management modules are designed, developed and implemented by software engineers/architects. This tool is designed to support the data sharing collaborations across multiple institutes and can be managed by an individual principle investigator. Access to the data entry application is password protected and controlled by user authentication process and manager-controlled user management module. All cases entered into the virtual biorepository are based on interview data and the associated tissue/ biopsy data for the entire participant.

### 

#### The work flow for entering the data into the web-based data entry tool

**Figure 4:**
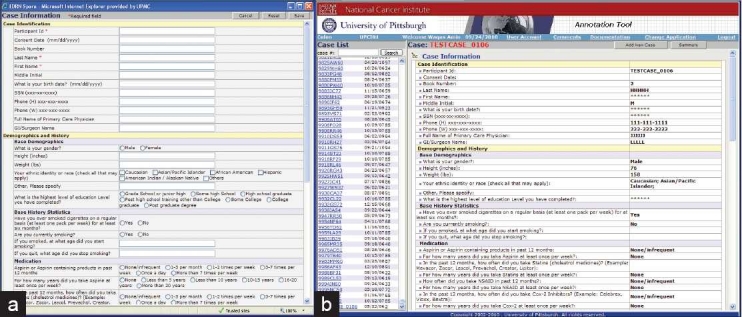
(a) The Early Detection Research Network (EDRN) colon data entry application that allows honest brokers to enter data on web-based data entry interface. (b) The EDRN colon data entry application

The multimodal datasets that are collected from different clinical/research sources are entered into the databases manually by using the data entry tool. This tool is password protected and is accessible. The HSTB identifies cases appropriate for inclusion in the Colorectal and Pancreatic Neoplasm Virtual Biorepository. There are three local tissue banks in the UPMC network, including UPMC Presbyterian, UPMC Shadyside and UPMC Magee Women Hospital. The local tissue bank preprocesses data on these cases. The handling of the specimens and the preprocessing of tissue banking data is the responsibility of the committed tissue bank technician. The most important component of preprocessing is deidentification. All deidentification occurs at the HSTB banks. No identifiable data is sent to the virtual biorepository. Deidentified data are entered into the database through a web-based data entry tool. The data entry web site uses radio buttons, combo boxes and other highly constrained data elements. The local banks label each case with a deidentified number. This number is used to link the information in the warehouse to the cases in the HSTB. The linkage codes are stored locally, using appropriate electronic and physical safety measures. Only the local banks have access to these linkage codes. The warehouse contains very minimal demographic data and complies with all HIPAA requirements. Cases entered into the virtual biorepository are scanned for logical errors (e.g., first recurrence before diagnosis, etc.) [Figure 4 a–c].

### Database Access Levels

**Figure 5:**
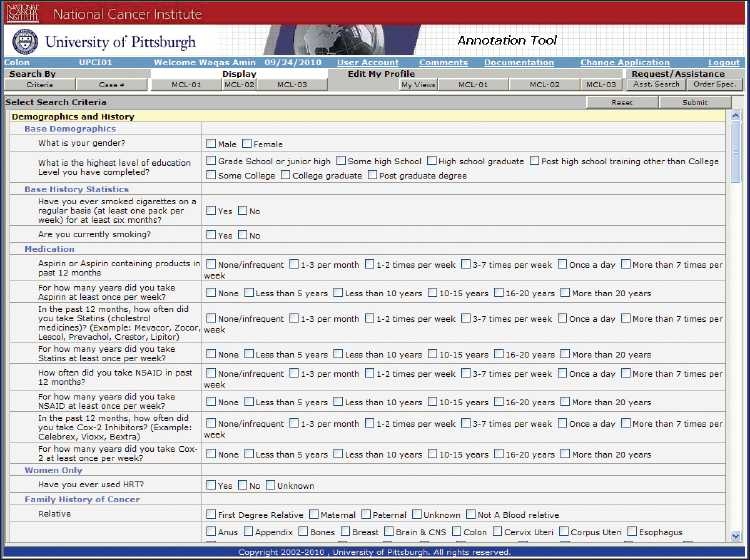
The early detection research network colorectal and pancreatic virtual biorepository query tool criteria selection page: users can select the specific criteria for searching the central database

**Figure 6:**
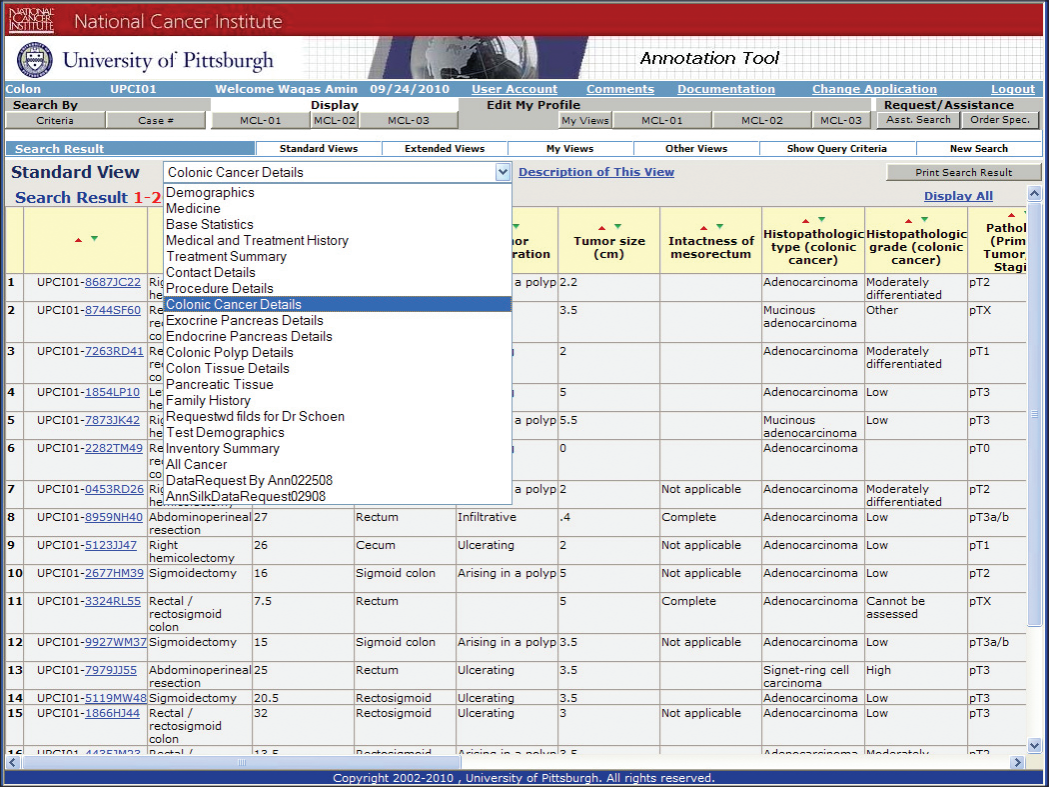
Approved investigator query tool: the approved investigator query tool shows a detailed annotation of the cases available. Users of this level can view data in multiple predefined views

**Figure 7:**
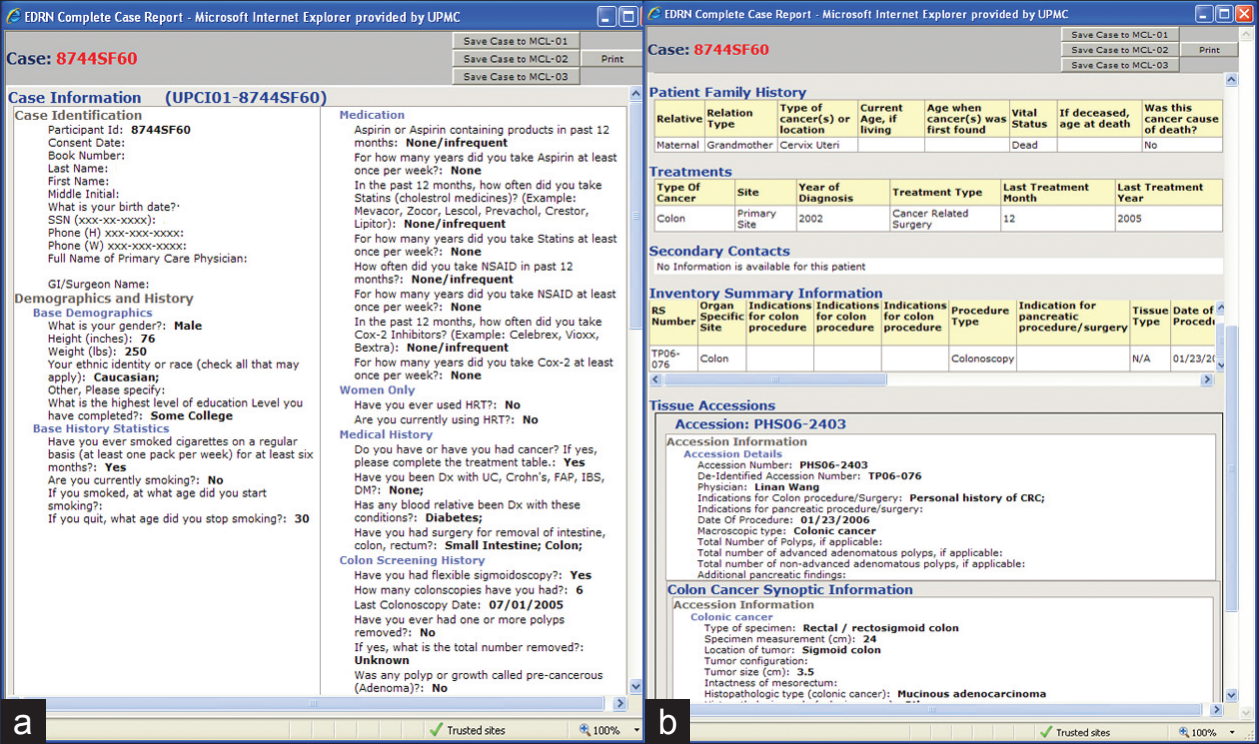
(a–b) Detailed deidentified clinical data of an individual case that is accessible to an approved investigator

**Figure 8:**
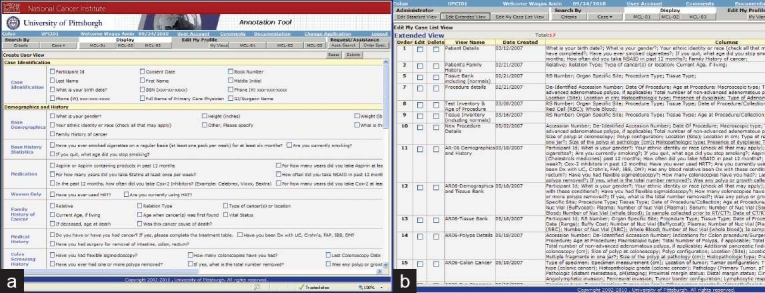
(a–b) Users with administrative rights that can customize their own data view and description but also edit and customize the “standard” and “extended” views for the rest of the user community

Initially, access is limited to members of the EDRN Colon Working Group using a user name and password system. The data administrator can provide user name and password for approved researchers or data mangers viewsto access the tool. The query tool access to the central database is through a highly constrained “click and point” interface. The data in the database allows queries on approved data elements and is also based on the researcher’s IRB approval. The specificity of the data returned will depend on the user’s profile. Since April 2003, a total of 12,384 queries were made in the database and 7,206 user session hits were recorded. There will be two user profiles as follows:

1. Approved investigator query tool is a password-protected tool, which will be distributed to those research investigators who have approved research protocols within the EDRN Colon Working Group. It will allow users to refine and compile case lists for their application and also to mine and modify the data set on the cases where they have received biospecimens. The query tool provided search on the all the annotated data associated with each subject through multiple predefined standard views of the data set and also allowed users to customize their own views and save under their account [Figures 5–7].
2. Data administrator query tool is a password-protected tool, available only for the internal data administrators. It is meant to be used by data administrators to address QA issues regarding the data. The main difference between this view and the approved investigator tool is that this tool allows the user to search by “all Subjects” or “limited subjects” based on consent for a particular study [Figure 8 a and b].

### Data Integration with Other Data Resources

The EDRN Colorectal and Pancreatic Virtual Biorepository database supports the integration with other prominent clinical/research data sources of patient-related information regarding diagnosis, follow-up, treatment and outcome. The pathology annotation and inventory data of banked tissues are tracked by the biorepository team by using the synoptic reports from the anatomic pathology information system. The tumor registrar and data manager gather follow-up and outcome-related data by reviewing medical records. This allows the users of this biorepository to query the complete case and have the overall view of patient progress and history.

## DISCUSSION

Experts from various fields have dedicated considerable time and energy to develop a biospecimens resource with high-quality annotation. Professionals from various disciplines including pathologists, gastroenterologists, informaticians, researcher specialists, cancer registrars and biostaticians contributed their work and efforts, which is integrated in this initiative. In a clinicians’ domain, they focused on developing CDEs that are aimed at gathering information based on current standards of clinical, pathology, follow-up (protocols) and cancer outcome. They also had to consider a future projection of at least 5 years during which any data may become clinically significant. This prospective data collection was also included in CDE development/structure. Similarly, the research scientists were responsible for insight into critical factors involved in recognition of risk factors, diagnosis, prognostication and treatment of colorectal and pancreatic cancers. The resultant data sets will hopefully cater to the needs of the research community and be the basis for enhancing future translational research activities. Data gathering is the responsibility of cancer registries, research nurse coordinators and tissue banker. In CDE development, they provided valuable input regarding metadata and data types.

Moreover, the definitions of the CDEs and their associated Meta data need to be clearly understandable to all those who collect data. Through the use of ISO 11179 compliance standards, the goals of collecting annotation data of high quality were achievable.[15] Although the concept of formalized Meta data is fairly straight forward, it has been rarely incorporated by clinical and research groups building databases. Database developers and informatics specialists developed the architecture to incorporate the CDEs to a database and they worked out the technical issues.

The integration of CDEs from multiple data sources within a database that are ISO compliant will allow interoperability and standardization of research data that will be easily understandable and can be implemented across multiple institutions. This will exponentially increase the statistical power of the research conducted, also allowing us to follow upcoming trends and issues in cancer research. It is critical that these trends and issues be addressed if we are to find methods to reduce the cancer burden and cancer pain and suffering, as is the ultimate goal of the NCI.[16] The value of tissue banks and the informatics that support these goals are clearly outlined in the NIH and NCI strategic roadmaps.[17]

### Annotation Process and Deidentification

In the process of annotation, all relevant clinical, cancer registry and pathologic information was manually entered into the database via a data entry web interface. This entry process was strictly integrated, deidentified and standardized. By integration, we mean a process of following and collected data from multiple clinical systems over time regarding a selected patient. For instance, gathering data from different sources like the pathology information system, tissue bank inventory system and cancer registry. There are two major obstacles in this process. The first is the correct identification of a certain patient in multiple systems or hospitals and the second is pinpointing only applicable information. In order to track follow-up data on patients who use multiple health care systems, one must be able to incorporate the patient data from various sources. This can be achieved by common linking patient identifiers or health information; nevertheless, discrepancies can occur owing to errors based on data entry issues or the absence of specific identifiers unique to the patients. The EDRN Colorectal and Pancreatic Virtual Biorepository initiative addresses these obstacles by a process of manual data collection and entry by the data managers. This ensures that the focus on data quality is maintained. The collected data that has a temporal relationship is carefully evaluated for context before it is captured into the database. We hope to overcome such challenges in the future using automated data retrieval through electronic queries of existing systems (i.e., Electronic Medical Record and the Enterprise Tumor Registry). These would require the use of messaging standards (i.e., HL7 and DICOM) and vocabulary standards (i.e., SNOMED and UMLS).

Deidentification involves removal of clinical identifiers from clinical data to make it available as deidentified for research use. This process is undertaken by honest brokers that are authorized to view the identified data and integrate the data sets with research numbers and then proceed to deidentify the data, making it available for research use. Honest brokers are authorized to view the identified data, collect clinical data, categorize the integrated data sets and then conceal the clinical identifiers before the integrated data is made available to the investigators. The database then assigns a random 10-digit deidentification number for a particular case. The link between this deidentified case number and the actual patient identifiers is maintained in strict privacy by a built-in functionality of the database, allowing the refreshing and QA of data exclusively by the honest broker. At no point are the patient identifiers ever made viewable on the public or investigator query interface. With the deidentification process, clinical follow-up and outcome data can be added via the deidentified code rather than using the patient’s name or medical record number.

In this project, standardization is ensured, which means that data is collected and implemented in a uniform manner and the resultant data sets are all understood exactly the same. In this initiative, standardization was achieved by (1) redesigning clinical systems (for capturing synoptic pathology reports) and (2) accepting clinical data elements into the central database (treatment and follow-up data from the Tumor Registry). Quality assurance and quality control protocols were implemented at all the three levels: (1) data transmission, (2) clinical/follow-up data and 3) pathological data.

### Web-Based Query Tool

A query tool was designed using Oracle database platform as a layered web-based tool to share data between internal members as well as the IRB and Scientific Committee Review Board-approved researchers. This query tool boasts speed and high security as well as expansion capabilities for incorporating new data elements or integrating existing system features. Emphasis has been placed on the ability to define user access level to the data sets. Patient privacy is of utmost importance and we also provide functionality for data monitoring.

## CONCLUSION

At present, there are increasing numbers of national and statewide efforts to promote the development of tissue banks and also to encourage these tissue banks to share both tissue and data. Currently, many tissue banks such as the Cooperative Prostate Cancer Tissue Resource (CPCTR),[18] Pennsylvania Cancer Alliance for Bioinformatics Consortium (PCABC),[19] Cooperative Breast Cancer Tissue Resource (CBCTR),[20] Cooperative Human Tissue Network (CHTN),[21] Cancer Family Registries (CFR)[22] and Specialized Programs of Research Excellence (SPOREs)[23] involve multiple institutions. These virtual biorepositories differ in methods of data and tissue collection. However, the necessity for well-annotated tissues that can be reannotated with experimental data has driven many of these multi-institutional collaborations to develop standards of sharing data with other groups.

The synoptic template (CAP protocol and checklist and WHO standard) and the NAACCR core elements represent widely established data elements that are used (and often mandated) in many cancer centers. In this work, we have shown that the two representations can be combined to create a core set of clinical annotation for banked colorectal and pancreatic neoplasm specimens. The information collected for these data elements is carried out as a part of the routine hospital workflow so these data sets can be easily developed and sustained. The current set of elements are now running in a database system and we are considering mechanisms to establish these elements that are an ISO/IED-compliant data standard.

The EDRN database differs from most other tissue resources in that all accrued cases have undergone standardized pathology review and that the clinical data have been carefully collected using a standardized quality-controlled method. The resource has developed a highly effective informatics infrastructure that allows for efficient governance, standardized capture of data and detailed standardized annotation of cases across multiple cooperating sites. This infrastructure includes an operations manual, a histopathology guide and a database with common data elements for characterization of tissue samples and clinical follow-up data, and a QA process. The web-based query tool allows easy accessibility for IRB and SRCB-approved collaborators and researchers. Patient confidentiality is meticulously ensured. A dynamic campaign for marketing of the EDRN resource is currently underway. This includes a comprehensive brochure, an interactive website as well as an information booth at meetings and conferences.
